# Biological acidification of pig manure using banana peel waste to improve the dissolution of particulate phosphorus: A critical step for maximum phosphorus recovery as struvite

**DOI:** 10.1016/j.heliyon.2022.e10091

**Published:** 2022-08-12

**Authors:** L.B. Moyo, G.S. Simate, T. Mutsatsa

**Affiliations:** a School of Chemical and Metallurgical Engineering, University of the Witwatersrand, Private Bag 3, Wits, 2050, Johannesburg, South Africa; b Department of Chemical Engineering, National University of Science and Technology, Box AC 939 Ascot, Bulawayo, Zimbabwe

**Keywords:** Phosphorus, Struvite, Anaerobic digestion, Banana peel waste

## Abstract

Traditional disposal of agricultural bio-waste such as pig manure and banana peel waste poses an environmental nuisance. The uncontrolled disintegration of these waste materials decomposes to toxic effluent and methane a greenhouse gas twenty-one times more potent than carbon dioxide at trapping heat in the atmosphere, which is detrimental to the climate by elevating temperatures. Agricultural bio-waste is rich in nutrients that include nitrogen and phosphorus. Selectively separating these nutrients from the solid phase to produce high value products has been envisaged as an effective method of waste valorisation. This study aims to investigate the solubilisation of phosphorus (P) during anaerobic digestion (AD) of pig manure with banana peel waste as the co-substrate. The objective was to enhance the biological dissolution of the phosphorus from solid pig manure to the aqueous phase as this is envisaged to subsequently ease the recovery of P as a concentrated product via crystallization. Thereafter, phosphorus is used as a slow-release mineral fertilizer. Biological acidification was effective in reducing the pH to less than 6.50 from an initial pH of 7.28 at higher doses of BPW >100 g/L. Maximum dissolution of total phosphorus of 75% was observed at a pH of 5.40. Multiple regression analysis was used to correlate pH, banana peel waste concentration, and the anaerobic digestion time (ADT) to optimize the dissolution of P as this was deduced to be occurring at a low pH. A 2^nd^ order polynomial was deduced to best fit the data with an R^2^ value of 0.90. The p values for the HRT and banana peel waste concentration were both <0.05 showing that both variables had a strong influence on the pH.

## 1 Introduction

A perennial increase in demand for animal protein due to growth in urban and affluent populations has been evident in the past decades worldwide. The global animal protein market was worth $44.08 billion in 2019 and is expected to reach 58.50 billion by 2027 Research, 2022. This is owing to a growth in demand for proteins as nutritional and functional additives, as well as an increase in the use of animal protein in the food and beverage industry. However, strict restrictions governing the origin of food additives, as well as rising demand for plant-based proteins, have hampered the expansion of the animal protein sector. The introduction of protein supplements into previously unexplored markets, on the other hand, is expected to open up new markets in the near future. According to (Fortunebusinessinsights, 2022) pork products are a leading revenue contributor in the animal protein market owing to a rise in pork consumption with a 29.01% market share. Pig production has risen as a result of increased red meat prices and the fact that pig farming is more environmentally friendly than cattle farming because it causes less harm to grazing land and does not necessitate more land for pastures (The Patriot, 2021). The surge in processed pork consumption has resulted in an increase in pig manure production, necessitating effective waste management. However, getting rid of animal manure is a costly procedure that requires labor, raw materials, logistics, and available land. Furthermore, there are several environmental concerns linked with the disposal of such trash, as it may contaminate the air, water, and ground. For instance, pig manure in landfills can disintegrate to produce methane which is more than 21 times more potent than carbon dioxide at trapping heat in the atmosphere resulting in elevated temperatures. Due to irregular beef output and shortages over the decades, as well as an increase in indigenous pork producers, pig production now accounts for almost 29% of the local meat market in Zimbabwe, up from 8% in 1987 (The Patriot, 2021). Furthermore, in June 2019, subsidies from the European Union (EU) were used to help Zimbabwe's pig industry move up from subsistence to commercial status. As of January 2020, 1000 farmers in the Mashonaland West and East regions will profit from this initiative. So far, 734 farmers have signed up for the campaign.

Land spreading of waste material on farmlands is currently the most used method for pig manure waste disposal, which is an ineffective method of dealing with waste material. Pathogen buildup, low nutrient status, long composting time, mineralization, and odor creation are all disadvantages of land-spreading pig manure. Furthermore, incorrect waste management as a result of pig farming activities has been demonstrated to promote eutrophication, which is caused by nutrient excess in waterways, necessitating the improvement of waste management methods (Latif , 2015). The insufficient and/or incorrect handling of the aforementioned pig manure has necessitated the urgent improvement of pig manure management techniques. More particular, techniques for the timely usage and valorisation of pig manure as a measure of agricultural sustainability are required (Hemidat et al., 2022). Sustainable waste management is critical because it enables efficient economic growth while reducing negative environmental impacts (Ye et al., 2011). In this regard, a large number of researchers are actively researching novel solutions that would satisfy the public without jeopardizing the environment. The pig business is an example of a targeted industry where sustainable waste management solutions are particularly critical to fully realizing a circular economy. Anaerobic digestion is a well-known biological processing technology that has long been acknowledged as a long-term treatment method for biodegradable materials like pig dung (Achinas et al., 2019). Microbes drive anaerobic digestion, which is a multiphase, complex biochemical process. The four different processes of anaerobic digestion are hydrolysis, acidogenesis, acetogenesis, and methanogenesis (Magdalena et al., 2019).

In each phase of the anaerobic digestion process, several microbial groups participate, each with its metabolic behaviour. The acidogenesis phase is the most important of these four steps in determining the biomass conversion pathway. The recovery of nutrients such as nitrogen and phosphate is facilitated by anaerobic digestion. The technique concentrates nutrients in the liquid phase while also producing high-value biogas that can be used as a source of electricity. Moreover, anaerobic digestion mitigates a wide spectrum of environmental undesirables, improves sanitation, helps in air and water pollution control and reduces greenhouse gas emissions.

At the moment, a developing paradigm is such that digestate from anaerobic digestion can be further processed to produce a concentrated fertilizer rich in phosphorus known as struvite (NH_4_MgPO_4_.6H_2_O) which is a slow-release fertilizer (Piveteau et al., 2017). This mineral fertilizer is attractive as it is citrate-soluble and the slow nutrient release prevents soil tie up for maximum efficiency. In addition, it has been proven to reduce nutrient runoff thus, improving sustainable agronomics. In the recent past, various phosphorus recovery techniques from municipal wastewater have been widely researched and this has been commercialized by companies such as Ostara in Canada, although this has not been implemented at large scale for pig waste (Piveteau et al., 2017).

It's also worth noting that phosphorus is a critical component of life, as it's a necessary component of all living things and a nutrient in food creation. It's also worth noting that the method of recovering phosphorus from pig dung isn't well understood, as anaerobic digestion of pig manure has primarily been used to recover energy. In this difficult environment, however, collecting nutrients like nitrogen and phosphate from animal excrement has proven to be a viable procedure. However, recovering a significant amount of phosphorus from a slurry of pig manure, for example, necessitates an efficient dissolution procedure that allows phosphorous to be mass transferred from the solid waste into the liquid phase for easy precipitation or crystallization. At a pH of 5.50, up to 70% of total phosphorus in the solid organic waste can be dissolved, according to previous research. This means that phosphorus recovery can be improved by using acidic dissolution, solid-liquid separation, and then increasing the pH of the liquid phase.

The use of inorganic acids such as hydrochloric acid has proved to make the process of recovering phosphorus from solid organic waste less economically feasible due to high maintenance costs as a result of corrosion and having a relatively negative impact on the environment as it increases salinity as well as safety concerns when handling the acids. Consequently, for the process to be cost-effective and environmentally sustainable, modifying the acidification process is imperative. One way of achieving this is through the acidogenesis process during anaerobic digestion which enables the natural acidification of substrates under anaerobic digestion.

It should be noted, however, that employing feedstock as mono substrates have been one of the difficulties in assuring good nutrient solubility during anaerobic digestion. Due to nutritional imbalance and a lack of a diverse variety of microorganisms required for anaerobic digestion, this is detrimental to the efficiency of the hydrolysis and acidogenesis processes (Gu et al., 2018). Previous research has shown that anaerobic co-digestion of two or more substrates is a viable option for mitigating the disadvantages of mono-digestion. Due to the synergistic effect of the materials utilized, anaerobic co-digestion will be advantageous in ensuring nutritional balance. Furthermore, it speeds up hydrolysis, which is the rate-limiting stage in anaerobic digestion. It has also been demonstrated that a two-stage anaerobic digestion process of fruit and vegetables mixed with pig dung at a 70/30 w/w ratio results in an acidogenic stage pH of 4.2, a pH at which phosphorus is largely dissolved in pig slurry (Piveteau et al., 2017). Inherently, biological acidification of pig manure with organic co-substrates might be considered as the first step in a bioprocess targeted at nutrient recovery, including nitrogen and phosphorus, as well as bioenergy recovery (methane).

This study attempts to use banana peel waste as the organic co-substrate to enhance the biological acidification process. Banana peel waste has a substantial amount of micronutrients which are essential for micro-organisms metabolism during anaerobic digestion with phosphorus (211.3 ppm), magnesium (44.5 ppm), and potassium (4.39 ppm) (Hassan et al., 2018). In addition, Banana peel waste constitutes a significant amount of soluble sugars which include glucose, sucrose, and fructose as well as organic acids which include (Serna-Jimenez et al., 2021). Characterization of the dry matter of banana peel waste has shown that soluble sugars can constitute 38-45wt% (Happa Emaga et al., 2007). These sugars can readily be converted to organic acids during AD to aid the acidic dissolution process of nutrients (Magdalena et al., 2019).

Besides, banana peel waste is also rich in micro-organisms which are imperative in the conversion of various compounds to acidic compounds required to aid the dissolution process (Liang et al., 2016). The objective of the study is to deduce a relationship between banana peel waste, pH, and hydraulic retention time which are critical variables in the anaerobic digestion process to ensure maximum dissolution of phosphorus as well as to deduce the extent of dissolution of nutrients required for struvite precipitation when pH is reduced.

## 2 Materials and methods

### 2.1 Preparation and characterization of banana peels

Banana peels were collected and cut into small pieces. Thereafter, the small pieces were washed with distilled water to remove any external dirt. The banana peel waste (BPW) was then placed in an oven and dried for a period of 24 h at 90 °C. The dried banana peels were then finely grounded using a mortar and pestle. The ground samples were sieved and graded into small particle sizes of less than 250 μm.

### 2.2 Biological acidification of pig slurry in batch tests using banana peels as co-substrate

The raw pig slurry was collected from a commercial scale pig farm in (Bulawayo, Zimbabwe) with the composition shown in Table 1. One litre vessels were filled with 640 g of raw pig slurry and six different dosages of treated banana peel waste were used (20, 40, 60, 80, 100, 120 g/L-slurry, in named reactors (R1, R2, R3, R4, R5, and R6 respectively) in duplicate. An additional bottle inoculated with pig manure slurry only without banana peel waste was used as a control (R0). Thereafter, the bottles were sealed, the temperature was set at 37 °C in a water bath and periodic mixing was conducted every 12 h through manual shaking of vessels before sample collection. Samples were collected simply by removing the lid and pouring the liquid from the bottles. Sample collection occurred after 24, 48, 72, 96, and 120 h.

**Table 1:** Feed Characteristics (g/L) (concentrations expressed per litre of raw pig manure).

TSS	VSS	T-P	T-N	T-Ca	T-Mg	pH	PO_4_–P	NH_4_–N	Ca^2+^	Mg^2+^
63.8	34.6	1.96	5.87	3.75	2.22	7.28	0.068	3.25	0.32	0.17

### 2.3 Analysis

The pH was measured using an OAKTON 700 pH meter immediately after each sample collection. Total suspended solids (TSS), and volatile suspended solids (VSS) were measured in triplicates on the raw pig slurry with standard methods (APHA, 1998). Acid digestion of the pig manure ashes was carried out to determine the phosphorus, calcium and magnesium. About 200 mg of ashes were added to 0.5 g of K_2_SO_8_ and 5 mL of a mix of H_2_SO_4_/HNO_3_ (75:25) in triplicate. The samples were autoclaved at 110 °C for 1 h at a pressure of 1 bar. The concentration of phosphorus was measured using a standard method at the wavelength of 880 nm (expressed in gram per litre of raw pig manure in Table 1) using UV-1800 UV-V Spectrophotometer manufactured by the Shimadzu corporation. For calcium and magnesium measurement, the concentrations were determined by a flame AAS using a GBC XplorAA Dual at 422.7 nm and slit width of 0.5 nm and 285.2 nm and a slit width of 0.5nm respectively.

## 3 Results and discussion

### 3.1 Effect of banana peel waste concentration on pH and fermentation product in slurry

**Figure 1:**
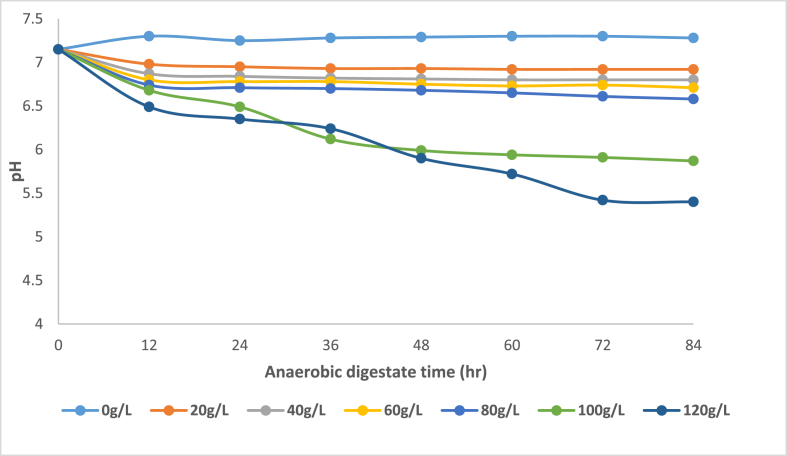
pH variation across time for each initial BPW concentration.

Figure 1 shows the pH variation with anaerobic digestate time (ADT) (average time interval over which substrate is kept inside the digester) for different doses of banana peel waste which are critical factors as micro-organism populations and enzymatic activity during anaerobic digestion is dependent on the parameters.

It was anticipated that the components of banana peel waste, coupled with those of pig manure, such as fermentable sugars will disintegrate with time to form volatile fatty acids and other organic acids which in turn reduce pH during anaerobic digestion. This logic was evident in R1 to R6 where there was a steep decrease in pH after 12 h as compared to the control R0 which did not show any statistically significant pH reduction during that period, instead, the pH was stable. This observation concurred with findings by (Piveteau et al., 2017). Considering a moderate dosage of banana peel waste represented by R1 to R4 after 12 h there was minimal change in the pH variation indicating that there was a deficiency of fermentable sugars available for the acidogenesis process to occur or a longer ADT time was required. On the other hand, in R5 and R6 there was a continuous decrease in the pH inferring that a higher dose of banana peel waste provides a substantial amount of fermentable sugars that could readily be disintegrated into organic acids. The lowest pH of 5.40 was achieved in R6 which had the highest banana peel waste concentration after an ADT of 84 h. However, at this high banana peel waste dosage, between 72 and 84 hs there was no statistically significant change in the pH which can be attributed to higher loading rates which mediate on inhibition of the anaerobic digestion process due to mass transfer limitations (Magdalena et al., 2019). In addition, after 72 h for R1 to R6 there was less variation in the pH, this could be due to the anaerobic digestion stabilizing. Consequently, the organic acid production in the hydrolytic and acidogenic is balanced with subsequent utilization by acetogenic and methanogenic micro-organisms which concurs with (Magdalena et al., 2019). In addition, according to (Yellezuome et al., 2022) at higher retention times ammonia formation is favoured from components such as proteins which take more time thus requiring high retention time to disintegrate. This improves the buffering capacity of the bio-digester resulting in minimal variation of pH.

### 3.2 Lactic acid formation

**Figure 2:**
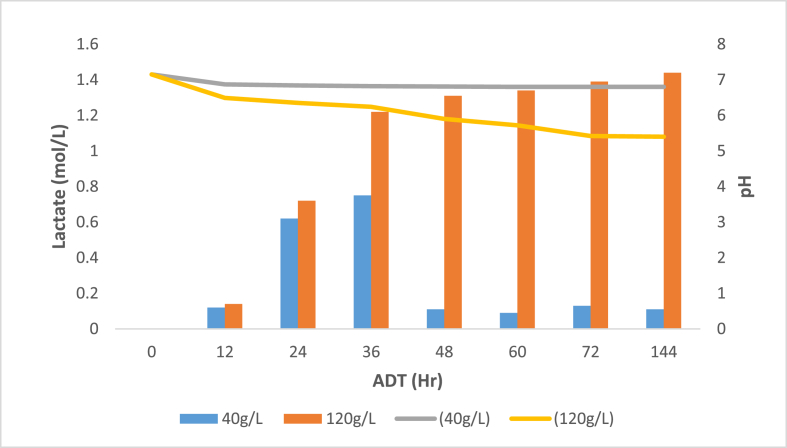
Lactic acid formation and pH values against ADT.

Acidifying bacteria convert water-soluble chemicals, such as hydrolysis products, to short-chain organic acids, such as lactic acid. The generation of lactic acid and the resulting pH fluctuation during the co-digestion of pig manure and two distinct dosages of banana peel waste of 40 and 120 g/L are depicted in Figure 2. At an ADT of 12 h, both dosages of 40 and 120 g/L produced less than 0.20 mol/L of lactic acid. This was owing to a lack of soluble organic material easily available to be degraded and converted to organic acids, as well as a lack of bacteria and archaea responsible for organic matter digestion. Moreover, the acidogenesis step is dependent on the hydrolysis phase to occur initially which is the rate-limiting step that mediates the minimal formation of organic acids (Yadav et al., 2022).

Figure 2 demonstrates that at a low banana peel waste concentration of 40 g/L, the pH fluctuated somewhat but remained steady around neutral. This indicates that there was a scarcity of soluble organic materials that might be converted to lactic acid quickly. Figure 4 also demonstrates that the amount of lactic acid generated and the pH was interrelated, with an increase in lactic acid synthesis resulting in a decrease in pH. As can be shown, with high banana peel waste concentrations, the pH dropped dramatically from an ADT of 0–12 h, indicating a faster hydrolysis and acidogenesis process in R6 that resulted in a significant amount of lactic acid production (Lv et al., 2007). The fact that lactic acid has a low pKa value of 1 contributes to the initial intense acidification (3.86). It's also worth noting that the different types of microorganisms involved in the anaerobic digestion process have different duplication times: acidogenic microorganisms take about 30 min to duplicate, acetogenic microorganisms take 1.5–4 days, and acetoclastic methanogens take 2–3 days to duplicate. Acidogenic bacteria have a short duplication time, according to (Gomez-Quirega., 2022), which ensures that there are enough bacteria to break down organic substrates into organic acids. Because the fermentation conditions in the batch reaction were controlled and the system remained hermetically closed without interference from external sources, the microorganisms were also able to survive. As a result, the indigenous microbiota was able to metabolize sugars more efficiently, leading to increased lactic acid generation. Following a 36-hour ADT, the lactic acid concentration did, however, decrease at a dosage of 40 g/L. This could be due to lactic acid breakdown into propionic acid and acetic acid, which have lower pKa values of 4.87 and 4.76, respectively (Ranaei et al., 2020). Due to the existing buffering capacity in the reactor as a result of other pre-existing organic acids, there was no substantial change in pH. At the greatest ADT of 144 h, the highest lactic acid concentration (1.44 mol/L) was achieved, with the lowest pH of 5.40. This suggests that even though lactic acid has an onset of only two to three days it takes longer for the microbial community to stabilize ensuring a more robust process (Fan et al., 2021).

### 3.3 Magnesium, phosphorus, calcium and ammonia dissolution processes in pig slurry

**Figure 3:**
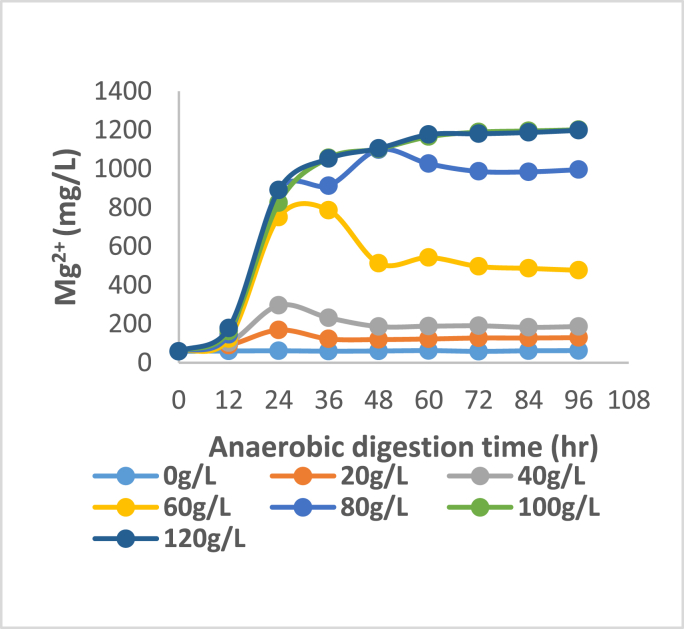
Dissolved magnesium at each BPW concentration.

**Figure 4:**
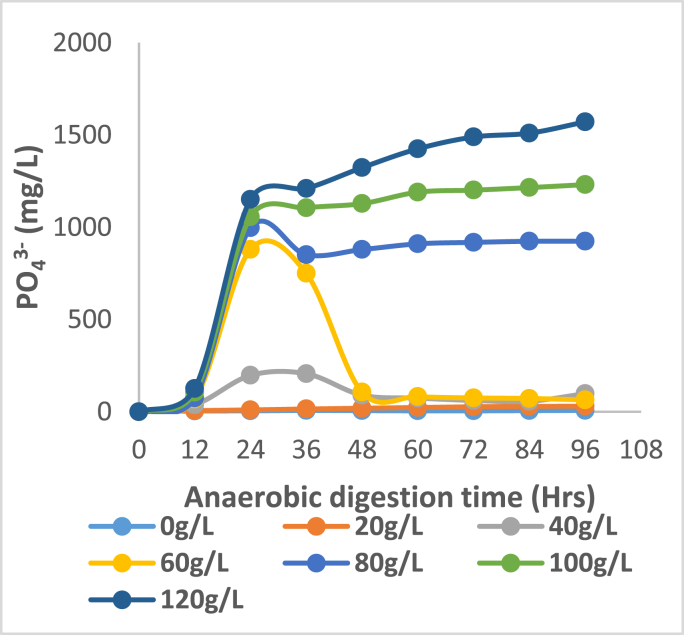
Dissolved phosphorus at each BPW concentration.

**Figure 5:**
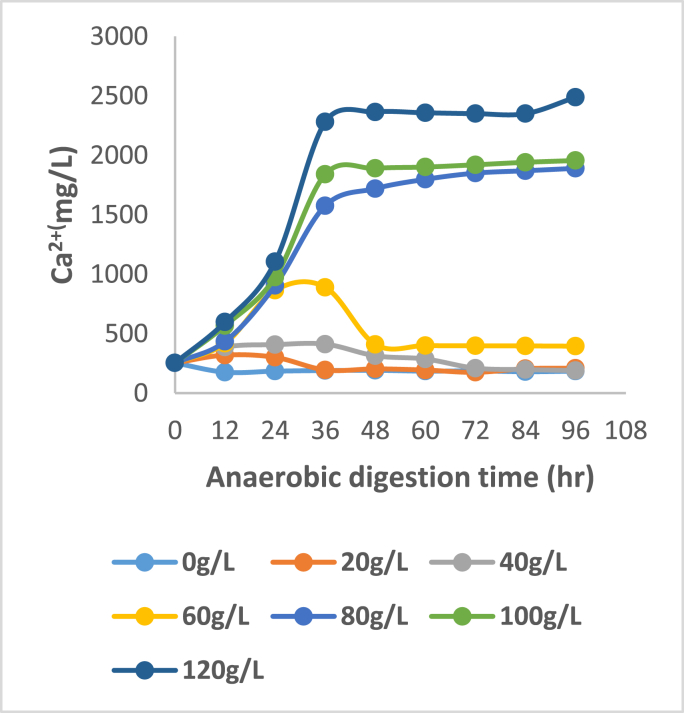
Dissolved calcium at each BPW concentration.

**Figure 6:**
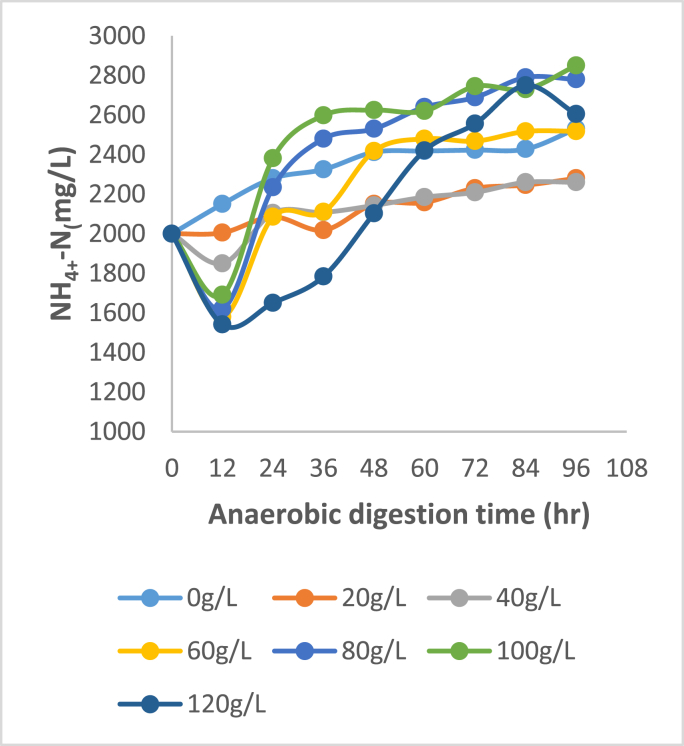
Dissolved nitrogen at each BPW concentration.

Figures 3, 4, 5, and 6 show that key nutrients such as magnesium (Mg), phosphorus (P), calcium (Ca), and nitrogen (N) associated with organic matter are released into the digestate as a result of biological acidification during anaerobic digestion. The maximum dissolution concentrations were 1198, 1571, 2488, 2850 mg/L respectively. Juxtaposing Figures 4 and 5 shows a very similar trend in the dissolution of P and Ca for almost all concentrations of banana peel waste, this may be due to the release of these ions being related to the dissolution of brushite Ca(HPO_4_)2H_2_O and hydroxyapatite Ca_5_(PO_4_)_3_OH which have Ca and P in common (Hilger et al., 2020). Whereas the dissolution of Mg shown in Figure 5, is predominantly dependent on the presence of struvite (MgNH_4_PO_4_.6H_2_O) and newberyite (MgHPO_4_.3H_2_O), the maximum concentration of Ca and Mg dissolved was 66%, and 54% respectively. There was a slight decline in the initial concentration of Ca and Mg at a dosage of 0 g/L this might be due to micro-organisms utilizing these secondary nutrients for growth, stimulating micro-organism activity and metabolism (Dos Santos Junior., 2021). Whereas, that of phosphorus was 75% the higher dissolution of Ca than Mg reflects that phosphorus was mainly bound to Ca in the bio-solids since they are both divalent (Mehta and Batstone, 2013). Moreover, in all scenarios investigated, there was a substantial amount of nutrients that were solubilized considering that during solid waste digestion only 50% of organic compounds undergo biodegradation. Whilst the remaining part of compounds remain in the primary state because of a lack of microorganisms participating in the degradation (Shah et al., 2014). The affinity of organic acids to dissolve the nutrients is also dependent on the biodegradation of the solid pig manure as there are mass transfer limitations if the pig manure remains in solid form. There will be minimal diffusion efficiency of the organic acid particles and adsorption of these particles onto the mineral-bearing compounds. The dissolution of phosphorus indicates a correlation with pH variation shown in Figure 1 where the lowest pH realized was observed in R5 and R6 which have a higher banana peel waste concentration this is where the maximum dissolution was realised. At low banana peel waste concentrations in R1 and R2 there was a statistically insignificant change in the Ca and Mg dissolution with concentrations averaging 220 and 92 mg/L respectively as shown in Figures 3 and 5. This could be attributed to that in R1 and R2 there was a minimal reduction in pH, consequently, this did not favour the dissolution of Ca and Mg as the aforementioned Ca and Mg orthophosphates are stable at a pH above 6.50.

From an ADT of 0–36 h, there is a slight increase in Mg, P, and Ca at modest banana peel waste concentrations in R3 and R4, influenced by a drop in pH; thereafter, there is a decrease in the aforementioned concentrations, which may be due to recrystallization of precipitates bearing these compounds as the pH has stabilized. In the case of phosphorus, the high extent of dissolution at the lowest pH was >90%, with a peak of 1571 mg/L, implying that a significant amount of phosphorus was entrapped in solid form in the feedstock. The high phosphorus following dissolution indicates that phosphorus-bearing compounds are amenable to organic acid dissolution and that the portions bound in the non-degradable fraction are amenable to organic acid dissolution. At the highest retention time of 96 h and highest banana peel waste concentration, the aforementioned maximum values were all recorded. This is due to improved hydrolysis of components like starch in banana peel waste to fermentable sugars, which is the first step before acidogenesis. In this respect, a short retention time prevents the hydrolytic bacteria from degrading the substrate, resulting in lower organic acid outputs due to less soluble organic matter available. The creation of organic acids, such as volatile fatty acids and lactic acid, requires this soluble organic materials. Inherently, a low pH does not result in reduced nutrient solubility, resulting in minimal nutrient dissolution (Cysneiros et al., 2012). A high banana peel waste concentration, as indicated in R5 and R6, results in high organic loading rates, which are harmful to the conversion of volatile fatty acids to methane because it leads to the accumulation of intermediary molecules such as volatile fatty acids, which lowers the pH. Furthermore, acidogens predominate at lower pH than methanogens, ensuring that fewer volatile fatty acids are converted to methane, resulting in the accumulation of volatile fatty acids and a lower pH while encouraging additional phosphorus dissolution, resulting in a fall in nitrogen concentration (Ahmad et al., 2020; Dahiya et al., 2015).

Sugar provided greater phosphorus solubility (>90%) for identical organic input rates (Piveteau et al., 2017). Because lignin limits the effective mass transfer of intracellular components that should be liberated and degraded into beneficial organic acids, this was not achieved with banana peel waste. The variation of nitrogen with ADT is shown in Figure 6. Initially, between 0 and 12 h, there is a gradual decrease in nitrogen, which could be due to volatilization of free ammonia during the anaerobic digestion process due to the degradation of ammonium as released as free ammonia as well as ammonium being used by microorganisms for growth (Möller and Müller, 2012). The source of nitrogen is largely in the form of proteins and amino acids, and the decomposition of these components creates ammonium, which is a long process that led to a gradual increase in the ammonium content as seen in Figure 6 (Goddek et al., 2018). Because a significant proportion of nitrogen is lost as free ammonia, the greatest dissolution of nitrogen achieved was 49 percent, which was the lowest recovery from the nutrients. High levels of ammonium in the reactor are toxic to methanogenic archaea, preventing the conversion of organic acids to methane, which encourages the formation of volatile fatty acids.

### 3.4 Statistical analysis

A correlation of pH with BPW concentration and ADT was deduced using multi-linear regression utilizing data from Figure 1 which is represented by the polynomial Eq. (1) relating pH, banana peel waste concentration and anaerobic digestion time. It was noted that the maximum phosphorus dissolution was observed at a pH between 5.4 and 6. Therefore it would be critical to deduce a relationship between the BPW concentration required to achieve a certain low pH for a given anaerobic digestate time (ADT). The highest R^2^ and adjusted R^2^ were 0.94 and 0.90 respectively determined from a 2^nd^ order model which was the best fit for the data. Table 2 illustrates the multi-linear regression data obtained.

**Table 2:** Multi-linear regression data for independent variables (Banana peel waste concentration, anaerobic digestate time, and pH as the dependent variable).

Model	RMSE	R^2^	Adjusted R^2^
**Custom Fit**	0.1644	0.9165	0.8887
**1st order**	0.2489	0.7875	0.7450
**2nd order**	0.1529	0.9439	0.9038

The polynomial equation relating pH, banana peel waste concentration and anaerobic digestion time that was deduced is as follows:

where x = banana peel waste concentration (g/L); y = ADT (hr) and f (x,y) = pH

(1) f (x,y) = 6.695 + 0.01142x+0.001826y+8.49e^−05^+-0.00021xy+8.266e^−05^x^2^y^2^

### 3.5 Potential for struvite crystallization

A high Ca^2+^ content was found in the digestate, which, with a molar ratio of Ca^2+^:Mg^2+^>1, could have a major impact on the quality of struvite produced, as Ca would compete with Mg ions for complexation and precipitation with phosphate species. The molar ratio of magnesium to calcium can be adjusted to optimize reaction kinetics while retaining struvite purity. To overcome this, an external Mg source, such as MgO, will be supplied, though it will be in excess. However, before adding the external Mg source, pre-treatment of the digestate can be done by precipitating Ca as CaCO_3_. The accumulation of volatile fatty acids as a result of inhibition of their breakdown due to high ammonium concentration improves the digestate's buffering capacity; struvite precipitation occurs more efficiently under alkaline conditions, implying that pH adjustment will be required. Although the pH can be slightly raised to activate microorganisms to break down the volatile fatty acids, this is not recommended. This reduces the buffering capacity, resulting in PO_4_–P concentrations of 200–400 mg/L in anaerobic sludge. Precipitation occurs at a lower pH with increased PO_4_–P and/or precipitant concentrations, resulting in lower operating costs. Struvite precipitation occurs in alkaline settings, yet optimal phosphorus dissolution occurs in acidic ones, necessitating additional chemical expenditures to overcome this. By removing carbon dioxide from the digestate before precipitation, which raises the pH, the chemical expenses added can be decreased.

## 4 Conclusions

Biological acidification of pig manure aided by banana peel waste is effective in the dissolution of particulate phosphorus. A relationship between pH, anaerobic digestion time, and banana peel waste concentration was deduced which can be used to deduce the optimal banana peel waste dosage and retention time to reach a low pH where the maximum dissolution of nutrients was observed. Furthermore, it was deduced that unlike sucrose which had high dissolution of phosphorus >90% this could not be achieved with banana peel waste due to its composition which contains components such as lignin which can hinder the mass transfer operations. Choosing the appropriate co-substrate and blend ratio will not only be effective in solubilising nutrients but will also enhance synergism, dilute disruptive compounds and improve digestate quality. Investigations on whether pre-treatment of banana peel waste may be effective in reducing the detrimental effects of such components may be required to be carried out.

## Declarations

### Author contribution statement

L.B Moyo: Conceived and designed the experiments; Performed the experiments; Contributed reagents, materials, analysis tools or data; Wrote the paper.

G.S. Simate: Analyzed and interpreted the data; Wrote the paper.

T Mutsatsa: Contributed reagents, materials, analysis tools or data.

### Funding statement

This work was supported by University of the Witwatersrand, Johannesburg.

### Data availability statement

Data will be made available on request.

### Declaration of interests statement

The authors declare no conflict of interest.

### Additional information

No additional information is available for this paper.
